# Frontal Cognitive Impairment and White Matter Hyperintensities in Probable Alzheimer’s Disease

**DOI:** 10.1002/alz70861_108219

**Published:** 2025-12-23

**Authors:** Mee Young Park, Jean Seo An

**Affiliations:** ^1^ Neurology Yeuengnam University Medical Center, Daegu Korea, Republic of (South); ^2^ UIC, 1200 West Harrison St., Chicago, IL USA

## Abstract

**Background:**

White matter hyperintensities (WMHs), often linked to cerebral small vessel disease and amyloid angiopathy, are frequently observed in Alzheimer's disease (AD). This study investigated whether the severity of WMHs is associated with differences in frontal lobe cognitive function and psychiatric symptoms in patients with probable AD

**Method:**

Fifty patients aged 50–90 with probable mild to moderate AD (per NINCDS‐ADRDA criteria) were assessed. Participants were divided into minimal (*n* =28) and moderate‐to‐severe (*n* =22) WMH groups using FLAIR MRI and the CREDOS scale. Cognitive function was evaluated using the K‐MMSE, CDR, and the SNSB‐II neuropsychological battery. Psychiatric symptoms were assessed with the Neuropsychiatric Inventory (NPI) and Geriatric Depression Scale (GDS).

**Result:**

• The moderate‐to‐severe WMH group was older (*p* =0.002) and had more women (*p* =0.04), but groups were similar in education and hypertension rates.

• No significant differences were found in global cognitive scores (K‐MMSE, CDR) or in any major cognitive domains.

• However, the Digit Span Forward test showed a trend toward poorer performance in the moderate‐to‐severe WMH group (*p* =0.06), suggesting possible frontal lobe vulnerability.

• Depression and neuropsychiatric symptoms did not differ significantly between groups.

**Conclusion:**

While global cognition and psychiatric symptoms were unaffected by WMH severity, a trend toward reduced frontal function was observed in patients with more severe WMHs. These findings suggest WMHs may contribute to subtle executive dysfunction in AD and warrant further investigation.

